# Body Size Judgments at 17 ms: Evidence From Perceptual and Attitudinal Body Image Indexes

**DOI:** 10.3389/fpsyg.2019.03018

**Published:** 2020-01-17

**Authors:** Ana Clara de Paula Nazareth, Vinícius Spencer Escobar, Thiago Gomes DeCastro

**Affiliations:** Laboratory of Experimental Phenomenology and Cognition, Institute of Psychology, Federal University of Rio Grande do Sul, Porto Alegre, Brazil

**Keywords:** body image, implicit cognition, perceptual awareness, experimental psychology, size judgment

## Abstract

Evidence related to temporal control for stimuli presentation of whole-body image is generally associated with attentional bias to ideal thin bodies. Few studies present evidence concerning whole-body stimuli recognition during fast visual exposure intervals. The aim of this study was to evaluate the accuracy and reaction times for the judgment of different sized body silhouettes presented at 17 ms in a non-clinical sample. Thirty-one participants were divided in attitudinal and perceptual body image groups based on Figure Rating Scale output and performed two experiments. First experiment assessed perception and the clarity of visual experience for human and non-human body stimuli at 17 ms. A general accuracy of 69.17% was registered with no differences between perceptual and attitudinal body image groups. These results indicated that the way participants perceive their own bodies does not influence the recognition of general visual silhouette stimuli. It was also observed that the clarity of visual experience is positively correlated to stimuli recognition accuracy. In the second experiment participants had to respond in a seven-point Likert scale if the presented image of body silhouettes were bigger, equal or thinner than their own bodies. Trials were divided in two blocks based on spatial rotation, half at 0° and half at 180°. General accuracy for body silhouettes recognition was 41.1%. Greater accuracy recognition for regular positioned stimuli was observed. Attitudinal dimension of body image was not a predictor of differential performance whereas perceptual body image groups recorded contrasting recognition performance. Distorted body image participants presented higher accuracy than undistorted body image participants, with greater accuracy to thinner silhouette figures. Women had significantly higher overall accuracy than men considering both experimental blocks. When comparing the cumulative accuracy curves across experimental trials, an exposure effect was registered only for the first experiment. Results showed that body silhouette stimuli were judged in a fast exposure interval with differential accuracy rates only for perceptual body image groups. Such evidence signals that conscious body image can be associated to implicit detection of visual human body stimuli. Future studies should further test how traditional explicit body image outputs perform within experimental approaches.

## Introduction

Body image has been systematically investigated through a set of experimental methods that include evaluation of size judgment and perceptual awareness paradigms. Research on the topic has its modern roots in the beginning of the twentieth century (DeVignemont and Alsmith, 2017). However, the emergence of systematic research is tied to the use of depictive methods such as silhouettes scales (Stunkard et al., 1983) or self-report scales assessing an attitudinal dimension of body image (Menzel et al., 2011). By attitudinal dimension, literature in the field generally refers to feelings of satisfaction or dissatisfaction a person has about their body size and shape (Cornelissen et al., 2019). Even though these evaluation strategies specify patterns of experience toward its own body, little was known of perceptual body image until experimental methods were applied to the investigation (Martel et al., 2016). Conceptually, perceptual body image is usually associated with the accuracy with which a person can judge their own body size dimensions (Mölbert et al., 2017). Research on body recalibration and proprioception (e.g., Rubber Hand Illusion – Botvinick and Cohen, 1998) helped to shape investigations on perceptual body image dimension. At the same time, evidence from the somatosensory cortex associated to body image perception (Longo and Haggard, 2010) made clear that perception of the body is not a binary process, in the sense that an individual has or not a body image conscious experience in a specific moment. Instead, old definitions of body image that supposed a continuous process of body perception throughout action control and sense of position in space were summoned (e.g., Schilder, 1999). Contemporary integrative models assuming a continuous process between implicit cognition to explicit self-referred perception of its own body have taken mainstream discussion on body image (e.g., Pitron et al., 2018). Such models can be traced back to inputs from Maurice Merleau-Ponty’s phenomenology (Merleau-Ponty, 2006), but have been revived in recent cognitive science by representational models of low- and high-level cognition (e.g., Longo and Haggard, 2012; Cardinali et al., 2016).

Research on body image has intensified over the past three decades driven by clinical studies that have identified body image distortion and dissatisfaction in different population. Such growth has been accompanied by innumerous instruments willing to assess body image (Kling et al., 2019). However, most of the available tools are based on a reflective concept, which targets a thematic body image consciousness. As pointed out in the scientific beginning of the field, body image comprises an extended process that is developed since the early age implying both an implicit body schema and an explicit body representation (Gallagher, 1986). Therefore, investigation should aim levels of consciousness other than explicit body image.

Even with the inclusion of experimental methods in body image research, the accessed dimension of perception is generally associated with the explicit consciousness of bodily stimuli under manipulation. In this respect, without controlling for temporal exposure of body stimuli, research in the field has difficulties to specify if what are under evaluation in perceptual body image indexes is the physiognomic recognition of stimuli or the pure size dimension judgments of the body. The emphasis on explicit aspects of body image may be highlighted by a specific methodological choice directed to assess conscious body stimuli on visual research (e.g., Barra et al., 2017; Leehr et al., 2018). This literature is more commonly associated with the presentation of face stimuli in the field of face perception aiming at the discrimination of emotions (Thompson and Wilson, 2012; D’Amour and Harris, 2017). Examples of research comprise Stoddard et al. (2017) research on the recognition of emotions and early signs of psychopathology, and Bedford et al. (2017) study on the typical development of emotion recognition in infants. On the other hand, literature associated to bodily silhouettes stimuli is traditionally linked to tasks of body appreciation without any temporal control. Few software manipulating body silhouettes applies psychophysical methods (e.g., Gardner and Boice, 2004) and seek to expand depictive research rigor. However, exposure to stimuli in software generally does not include temporal control for fast responses (Caspi et al., 2017). In this sense, without knowing or properly investigating timeframes associated to body size judgments, no assumption regarding continuity from implicit to explicit cognition can be well established.

Different research using body stimuli tend to vary visual exposure time according to specific aims of the investigation (Sekunova and Barton, 2008; Bonemei et al., 2018). However, a small number of studies have actually investigated fast presentation intervals for this kind of stimulus. Majority of studies applying temporal control are more focused in exploring responses to the size and spatial orientation of the presented body stimuli than to the effect that the applied time control for stimuli presentation has in the actual responses (Ferrer-García and Gutiérrez-Maldonado, 2008). There is little evidence to indicate the relationship between the perception of whole-body stimuli and the exposure intervals related to the processing of a specific stimulus selected by participants. Such disregard for temporal control may be explained by how part of body size estimation research theoretically refers to perception, as a process guided by semantic affective and cognitive toward the body (Smeets and Panhuysen, 1995). In this sense, evaluations aiming exclusively at explicit cognition.

Notwithstanding, evidence exists for experimental time control based research in clinical settings. For example, Rivolta et al. (2017) presented stimuli of body silhouette and neutral stimuli for 200 ms to a clinical group with difficulties on facial recognition expressions (prosopagnosia). Results showed that in addition to impairment of facial recognition, this sample also presented impairment in the recognition of faceless body silhouette stimuli. Similarly, Duncum et al. (2016) presented body stimuli, objects and scenes for 500 ms to a group of body image concern participants. The set of stimuli were randomly presented in regular and inverted orientation. Accuracy rates were higher in all conditions for the participants with lower rates of concern with body image. In relation to reaction times (RTs), participants who were more concerned with body image had a lower average RT for faces, bodies and objects when they were presented in inverted orientation. These studies exemplify the literature emphasis on time control for object recognition rather than the time limits for size judgments or to how explicit body image judgments are related to implicit recognition of the body.

There are studies demonstrating an association between dissatisfaction with body image and attention bias to idealized thin bodies (e.g., Joseph et al., 2016; Moussally et al., 2016), which could reveal a connection between explicit and implicit body image processing. However, these studies are only able to evidence bias differences between satisfied and dissatisfied groups from intervals around 500 ms above. In general, women who are more dissatisfied with their bodies have an attention bias toward idealized bodies in intervals around 500 ms. This decision making occurs in a time interval already associated with explicit processing of response selection. Little is known regarding satisfaction and dissatisfaction with body image from performance within tasks aiming initial processing of visual stimuli.

To initiate a debate over initial processing of bodily visual stimuli, beyond time control, visual detection *per se* must consider the degree of complexity of the presented stimuli. Complex stimuli tend to present more realistic and detailed feature patterns. Simple stimuli, in turn, present fewer variable properties, usually limited to two-dimension presentation (Lamberts et al., 2002; Palumbo et al., 2014). In terms of clarity of the visual experience, Sandberg et al. (2010) tested 12 intervals, from 16 to 192 ms, with gradual variations of 16 ms to understand when conscious experience starts to occur for simple stimuli. The stimuli presented by the authors were four geometric silhouette forms, later masked by the union of all silhouettes until the emission of the response. Results evidenced that in 16 ms the sample already had an almost clear experience of visualization for the whole set of stimuli, reaching clear experience from 96 ms.

For body stimuli, factors such as real images, the presence of multiple colors, the size of the image, the contrast ratio between the image and the background are aspects that increase its complexity. In such cases, the use of very brief time intervals may frustrate the aim to capture an individual’s ability to discriminate such stimuli. In this sense, body stimuli could be represented reducing visual feature variability by means of pictographing the stimulus in monochromatic tones, broadening the contrast between stimulus and background, and reducing the depth of the target stimulus. In these situations, a brief temporal control of visual exposure could be successful in discriminating implicit processing of bodily stimuli. For top-down theories, as instance, pattern recognition could be applied as an explanation of how explicit body image perception is related to implicit self-representation of the body. Evidence for temporal control in the presentation of body silhouettes stimuli is not sound in body image research. In this sense no conclusive remark, considering the set of evidence available, can be established for a minimum interval necessary for its recognition in clinical and non-clinical groups.

Considering the assumption of a continuous process between implicit and explicit body image perception (Pitron et al., 2018), the aim of the present study was to evaluate body size judgments in a non-clinical sample at a brief visual exposure interval (17 ms). Two experiments were proposed to first assess the participants ability to correctly discriminate body silhouette stimuli at 17 ms (Experiment 1) and secondly to assess the participants ability to judge body size silhouettes relative to their own body size dimensions at the same timeframe (Experiment 2). The first experiment was thought as a baseline assessment for stimuli detection and an evaluation of visual clarity experience level toward body stimuli. Based on previous results (Pessoa et al., 2005), we expected participants to correctly discriminate body stimuli without a strong visual clarity experience. The main focus, however, is if non-clinical subjects will be able to produce accurate body size judgments at 17 ms in comparison to their own body size. Our hypothesis for the second experiment is that body size judgment will be accurate only when the judged stimulus represents similar size dimensions as that of the participants own body. To explore the relation between explicit and implicit cognition the sample was divided in body image groups, based on an explicit body image scale, to analyze accuracy and RT data in relation to these group divisions. Our hypothesis is that explicit criteria for body image perception will predict differential accuracy rates in the second experiment with body image distorted participants presenting lower accuracy rates than the undistorted body image participants group.

## Materials and Methods

### Participants

Initially 37 participants were recruited in four local University campuses. Six participants were excluded from final analysis due to data loss or as a result of presenting clinical symptoms that could interfere in visual perception. Final sample consisted of 31 participants (19 women, mean age of 22.9 years, SD = 2.98). Participants Body mass index (BMI) average was 23.9 kg/m^2^ (SD = 4.06 kg/m^2^). Sample size is compatible with previous studies on visual perception in body image (e.g., Castellini et al., 2013; Forghieri et al., 2016; Bailey et al., 2017). All participants had normal or corrected-to-normal vision. The research protocol followed the ethical standards of Brazilian regulation for studies with human participants (Resolution 510/2016 of the National Health Council) and was approved by the University’s Ethical Committee (Registered Protocol Number: CAAE 87592718.3.0000.5334).

### Instruments

The following instruments were applied: Figure Rating Scale (FRS – Kakeshita et al., 2009), Body Shape Questionnaire (BSQ – Di Pietro and Silveira, 2009), DSM-5 Self-Rated Level 1 Cross-Cutting Symptom Measure – Adult (American Psychiatry Association [APA], 2013), and a socio-demographic questionnaire.

#### Figure Rating Scale

The Figure Rating Scale (FRS – Kakeshita et al., 2009) applied in this study is the Brazilian adaptation and validation of Stunkard’s FRS (Stunkard et al., 1983). The original instrument consisted of 18 silhouettes, nine for female participants and nine for male participants. The Brazilian version of FRS consists of 30 silhouettes, 15 silhouettes for female participants and 15 silhouettes for male participants. Each silhouette figure corresponds to an index of BMI variation. The difference between two sequential silhouettes corresponds to intervals greater than 2.5 kg/m^2^ variance between each silhouette. The BMI range for the 15-silhouette set representing each gender group begins at 11.5 kg/m^2^ and ends at 48.75 kg/m^2^. The Brazilian FRS expanded the BMI differences between the silhouette figures, considering the diverse body patterns of the Brazilian population. Instrument application consists of asking the participant (1) which silhouette figure best represents their current body and (2) which silhouette figure best represents the body they would like to have. The Brazilian FRS provides two scores: the perceptual body image index and the attitudinal body image index. Perceptual body image index is calculated by subtracting the silhouette chosen as the participants’ current body from the silhouette figure correspondent to their own measured real BMI. Attitudinal body image index is calculated by subtracting the silhouette figure chosen as the body participants’ would like to have from the silhouette figure indicated as their current body. The Brazilian version of FRS presented good reliability indexes regarding the perceived body (α = 0.92) and the ideal body (α = 0.86) judgments (Griep et al., 2012). In the present study participants had a perceptual body image index average of 1.4 (SD = 1.4) variation and 0.9 (SD = 1.5) average variation for the attitudinal body image index.

#### Body Shape Questionnaire

The BSQ is a self-reported questionnaire that inquiries about body image general dissatisfaction. The Brazilian version of BSQ consists of 34 items corresponding four different factors: (1) self-perception of body shape, (2) comparative perception of body image, (3) attitude concerning body image alteration, and (4) severe alterations in body perception. The score sum indicates the corporal dissatisfaction in four levels: (a) no concern, (b) mild, (c) moderate, and (d) severe (Cooper et al., 1987). Validity evidence of BSQ to Brazilian population shows good internal consistency (α = 0.97) and factorial structure similar to the original questionnaire (Di Pietro and Silveira, 2009). In the present study participants presented a BSQ average of 76.8 points (SD = 25.5), which is below the cut-off point of 110 points considered for body dissatisfaction in the Brazilian version of BSQ.

#### DSM-5 Self-Rated Level 1 Cross-Cutting Symptom Measure – Adult

The DSM-5 Self-Rated Level 1 Cross-Cutting Symptom Measure – Adult consists of a mental health questionnaire for adult population. 23 screening items evaluate the following psychiatric domains: anxiety, depression, dissociation, sleep disorder, personality functioning, suicidal ideation, mania, memory, repetitive thoughts and behaviors, psychosis, anger, somatic symptoms, and substance use. Each item investigates whether and how often the participant has noticed and been disturbed by the symptom in the last 2 weeks (American Psychiatry Association [APA], 2013). Considering the research purpose, participants were excluded from data analysis only when presenting symptoms in more than two domains or when psychosis, dissociation or substance abuse were singly present.

#### Socio-Demographic Questionnaire

The socio-demographic questionnaire provided socio-demographic data of the participants such as age, color, gender, self-reported laterality and also information regarding the use of medications, physical activities and if they perceived intense variation in their weight for the last 5 years.

### Experiments

Two experimental tasks were performed by participants. Both tasks were programmed in OpenSesame (version 3.1.9 – Mathôt et al., 2012). Stimuli were presented on a 25” widescreen computer screen (60 Hz), in black background, with a resolution of 2560 × 1080 pixels. Eighteen experimental files were prepared, nine for male participants and nine for female participants, one file for each silhouette size. All participants performed and successfully passed a task comprehension test before starting the experiment and sat at a constant 50 cm distance of the computer screen.

#### Experiment 1

The first experiment consisted of 90 trials randomly presented including six silhouette stimuli: two hominid silhouette stimuli (Australopithecus), two homo sapiens silhouette stimuli (male or female, depending on participant’s gender), one silhouette of a monkey and one silhouette of a broom. With the exception of the broom, the images used in the experimental task were taken from the PhyloPic database and handled in Photoshop CS5 software. Each stimulus was presented 15 times and were each exposed at a constant 17 ms interval. After the presentation of each target stimulus, an oval-shaped visual noise was presented, pixelated in a Gaussian format at 350p×, for a constant interval of 116 ms. This interval is based on previous studies on perception and awareness of simple visual stimuli (Pessoa et al., 2005).

**FIGURE 1:**
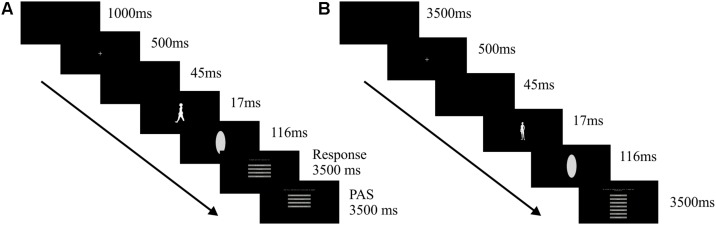
**(A)** Trial sequence for experimental task 1 and **(B)** trial sequence for experimental task 2.

Before each silhouette stimulus presentation, a fixation stimulus (white 4p× cross) was shown for a constant interval of 500 ms. After each silhouette stimulus participant informed by mouse response which image they had just seen, if “a homo sapiens,” “a monkey,” “a hominid” or a “broom.” A response timeout of 3500 ms was established for each trial. After the first response, a second response regarding the same trial was requested, in which participants should judge the clarity of his visual experience for each trial. A four point-scale of Perceptual Awareness Scale (PAS) was applied (Ramsøy and Overgaard, 2004). A response timeout of 3500 ms was also established for this response (Figure 1A). Unanswered trials for the first response were considered omission trials. This experiment had a self-controlled rest interval after 45 trials.

#### Experiment 2

The second experiment consisted of 280 trials randomly presented in two counterbalanced blocks. In one block silhouette stimuli were presented at a regular spatial orientation (140 trials), while in the other block stimuli were rotated at 180° (140 trials). Participants task was to estimate body silhouettes size in relation to their own body size. Seven images of the FRS were applied: the two extremes of the scale (extremely thin silhouette and extremely obese silhouette), a silhouette referring to the own real body size chosen by participant’s at the conventional FRS application before experimental tasks, two silhouettes immediately below the own real body size and two silhouettes immediately above the own real body size as informed by participant’s previously to the experimental tasks.

Each stimulus was presented 20 times at a constant 17 ms interval in a random order for each block. After the presentation of each stimulus, an oval-shaped noise pixelated in a Gaussian format at 350p× was presented at a constant 116 ms interval (Pessoa et al., 2005). After the stimulus presentation, a response screen requested participants to answer the similarity of the visualized silhouette figure in comparison to their own body. Accurate responses were only considered when participants matched the stimuli presentation with the stimuli description (e.g., much thinner stimulus within seven possible stimuli with “Much Thinner” response option). Accurate “Equal” responses occurred when equal response were given to the silhouette stimulus correspondent to the one selected as the participants’ current body in the pre-experiment FRS application. By means of mouse response on seven-point scale participants selected one of the following options regarding their own body size: “much thinner,” “thinner,” “relatively thinner,” “equal,” “relatively larger,” “larger” and “much larger.” The response screen was displayed for 3500 ms and participants were asked to respond as quickly as possible (Figure 1B). Unanswered trials were considered omission trials. Rest intervals were taken between and within the midpoint of each block.

### Procedure

Participants were invited to participate in the experiment by a pre-scheduled agenda at the laboratory room. Before conducting any research procedure, the participants were instructed on the objectives and ethical aspects of the research and were presented to the Informed Consent Form (ICF). In case of agreement to participate, the data collection procedures were initiated.

The data collection procedure was: (1) pre-test with the conventional application of the FRS; (2) first experimental task; (3) second experimental task; (4) response to the Socio-Demographic Questionnaire, DSM-5 Self-Rated Level 1 Cross-Cutting Symptom Measure – Adult and BSQ; (5) BMI measurement, performed with a stadiometer (Brand: WCS/Cardiomed) and a body weight balance (Brand: Plenna); and (6) closing conversation about the data collection. Data collection lasted approximately 50 min.

#### Group Formation

For the constitution of satisfied/dissatisfied groups and with distortion/without body image distortion, a FRS cut-off point based in previous experimental research was adopted (Pinhatti et al., 2018). Thus, participants who scored −1, 0 and 1 in the FRS, which corresponds to a variation of ± 5 kg/m2 (two times own silhouette figure variance) in relation to their BMI were considered satisfied and without body image distortion. On the other hand, participants with scores of −5, −4, −3, −2, 2, 3, 4, and 5 on both FRS scores were placed into the dissatisfaction and body image distortion groups, since they represent real body size (perception) and ideal body size (satisfaction) above ± 5 kg/m2 of variation in relation to their own bodies.

### Statistical Analysis

Experimental data was automatically transferred by the OpenSesame software to individual Excel worksheets (Microsoft) and then transferred to IBM SPSS (version 25) for statistical analysis. Descriptive analysis was carried out to observe the distribution of data normality. Trial response below 300 ms and above 3500 ms were considered invalid. Participants with an invalid trial rate above 10% were excluded from the study (Wang et al., 2012). Included participants in the study had an average of 1.07% (SD = 1.54%) invalid trials.

#### Experiment 1

The analysis of Experiment 1 consisted of three independent measures *t* tests for accuracy and RT as dependent variables between independent variables men and women, satisfaction and body dissatisfaction groups, and distorted and undistorted body image groups. Oneway variance analysis (ANOVA) considering stimuli class as independent variable were also performed to compare dependent variables accuracy and RT of the image discrimination and response time for visual clarity experience (PAS) response. The Bonferroni correction was used for this analysis. We also performed correlation analysis between BMI, BSQ scores, distortion and body dissatisfaction scores from the FRS and the task accuracy scores.

#### Experiment 2

The analysis of Experiment 2 consisted of three 2 × 2 mixed ANOVAs, considering dissatisfaction/satisfaction, distorted/undistorted body image groups, and men/women as intersubjective factors (one for each ANOVA) and block of normal presentation of the stimuli/block of inverted presentation of the stimuli as the within subject factor. Dependent variables for these analyses were accuracy and RT to the stimuli set. We also performed *t*-tests for independent groups taking distorted × undistorted body image groups and unsatisfied × satisfied body image groups as independent variables and accuracy means for each of the seven silhouette figures as dependent variable. Correlation analysis between accuracy, BMI, BSQ scores, distortion scores and body dissatisfaction from the FRS were also performed.

## Results

### Experiment 1

General accuracy rate for all figures was 69.1%. Specific ratings were 97.8% for broom, 74.9% for homo sapiens, 65.8% for monkey and 51.1% for hominid silhouette. No differences in performance were found between male and female participants [*t*(29) = 0.82, *p* = 0.936]. When considering satisfaction and body dissatisfaction groups, based on the FRS scores, group comparison did not indicate differences for the overall performance between groups [*t*(29) = −1.109, *p* = 0.277]. However, specifically for the identification of human figure, body dissatisfaction group presented a higher accuracy mean compared to the body satisfaction group [*t*(29) = −1.919, *p* = 0.041, *d* = −0.73]. In contrast, no differences were found between groups of body image distortion for this task [*t*(29) = 1.499, *p* = 0.145].

ANOVA testing for differences in accuracy, RT and RT for visual clarity experience between stimuli classes indicated a significant effect [*F*(3,120) = 14.973, *p* < 0.001, *f* = 0.647]. Bonferroni *post hoc* test evidenced that the broom stimulus was significantly more identified, and faster responded (RT and visual clarity experience RT) in relation to the other three stimuli classes. Homo sapiens stimulus was also more recognized than the hominid stimulus (*p* = 0.006, *d* = 0.78).

**FIGURE 2:**
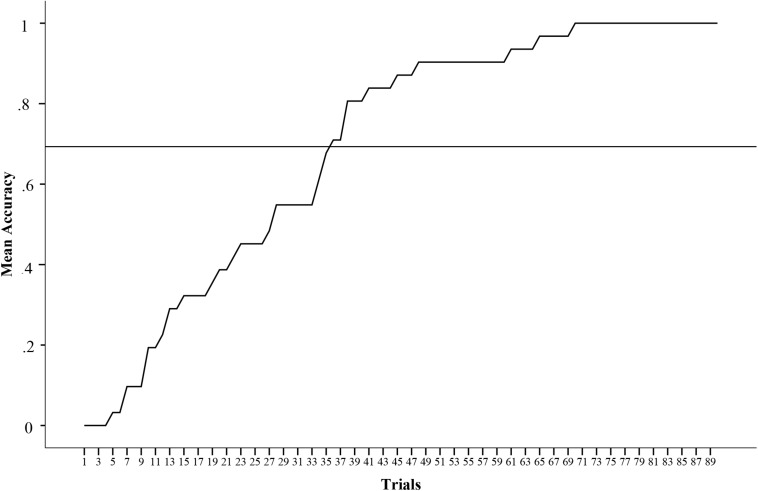
Accuracy average compiled for experimental task 1.

Correlation analysis evidenced a strong positive correlation between accuracy and visual clarity of stimuli measured by PAS (*r* = 0.86, *p* < 0.001). There was also a strong positive correlation between RT for stimulus identification and RT for visual clarity of the stimuli (*r* = 0.73, *p* < 0.001). Figure 2 presents the accuracy rate average throughout the trial sequence for this experiment.

### Experiment 2

#### Mixed ANOVAs for Body Image and Gender Groups

General accuracy rate for body silhouette figures was 41.1%. Table 1 presents body image and gender groups means and results for the mixed ANOVA’s. Comparison of the general accuracy rate between regular and inverted stimuli orientation blocks showed significant difference [*t*(29) = 2.528, *p* = 0.014, *d* = 0.64], with greater accuracy in the regular orientation block (*M* = 43.97%, SD = 8.86%) than in the inverted orientation block (*M* = 38.23%, SD = 9.03%). Gender differences were found with higher accuracy rates observed for women. A Mixed ANOVA revealed a main effect for both gender group and stimuli orientation block, but no interaction between these variables.

**TABLE 1:** Body image and Gender groups means and standard deviations with correspondent Mixed ANOVAs statistics for Experiment 2.

	**Regular**	**Rotated**	**Comparisons**	***F*(*df*)**	**η*^2^***
Body Satisfied	43.01 (2.1)	35.07 (2.1)	Satisfied × Dissatisfied	0.56 (1,29)	0.01
Body Dissatisfied	42.91 (2.5)	39.45 (2.4)	Regular × Rotated	12.53^∗∗^ (1,29)	0.30
			Interaction	1.93 (1,29)	0.06
Body Undistorted	40.75 (2.1)	33.15 (1.9)	Undistorted × Distorted	6.66^∗^ (1,29)	0.18
Body Distorted	45.66 (2.3)	41.48 (2.1)	Regular × Rotated	13.27^∗∗^ (1,29)	0.31
			Interaction	1.11 (1,29)	0.03
Men	37.67 (2.2)	32.20 (2.4)	Men × Women	10.85^∗∗^ (1,29)	0.27
Women	46.31 (1.8)	39.88 (1.9)	Regular × Rotated	12.52^∗∗^ (1,29)	0.30
			Interaction	0.08 (1,29)	0.00

*^∗^*p* < 0.05, ^∗∗^*p* < 0.001.*

Assumptions for the equality of variances and covariances matrices were all met for the performed Mixed ANOVAs. Regarding groups established based on the explicit body dissatisfaction criteria, a mixed ANOVA did not indicate general performance effect between groups, nor an interaction of the performance of the groups with stimuli orientation blocks. However, considering only the performance between blocks per group, an effect is observed, which is explained by a performance difference observed specifically for the satisfied group.

In turn, a mixed ANOVA based on body image distortion groups showed an effect of stimulus orientation on task performance and an effect of the distortion group on task performance. However, no interaction effect was observed between these variables for the mixed model. Both groups presented higher accuracy rates for stimulus recognition in the regular stimuli orientation block than in the inverted stimuli presentation. Also the distorted body image group presented a significantly higher accuracy index than the non-distorted body image group when considering all trials together [*t*(29) = −2.861, *p* = 0.008, *d* = −1.04].

#### Comparisons for Specific Silhouette Figures Accuracy

**FIGURE 3:**
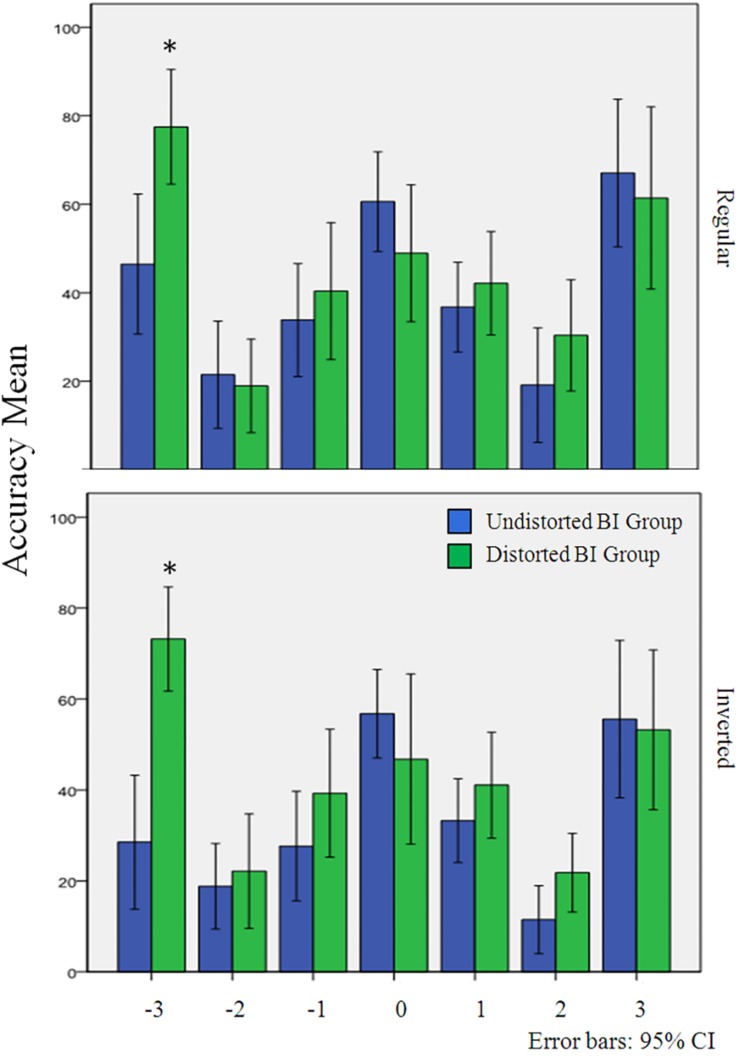
Accuracy averages of BI perceptual groups divided by silhouette figure and experimental condition in Experiment 2. Confidence interval of 95%. ^∗^*p* < 0.05.

In the analyses considering the explicit criteria for groups’ composition, a difference was found only for one silhouette figure between distorted and undistorted body image groups, as indicated in Figure 3. The distorted body image group had higher accuracy rate for the much thinner figure, both in the regular stimuli orientation [*t*(29) = −3.145, *p* = 0.004, *d* = −1.15] and in the inverted stimuli orientation experimental blocks [*t*(29) = −4.933, *p* < 0.001, *d* = −1.81].

#### BSQ, BMI and Reaction Time Analyses

Differences between satisfied and dissatisfied body image groups were only observed in the scores of BSQ dimensions “self-perception of body shape” [*t*(29) = −2.870, *p* = 0.008, *d* = −1.028] and “comparative perception of body image” [*t*(29) = −2.562, *p* = 0.02, *d* = −97]. Dissatisfied body image participants had higher averages in these scores. The mean BMI of dissatisfied participants was also higher than that of satisfied participants [*t*(29) = −2.484, *p* = 0.019, *d* = 1].

**FIGURE 4:**
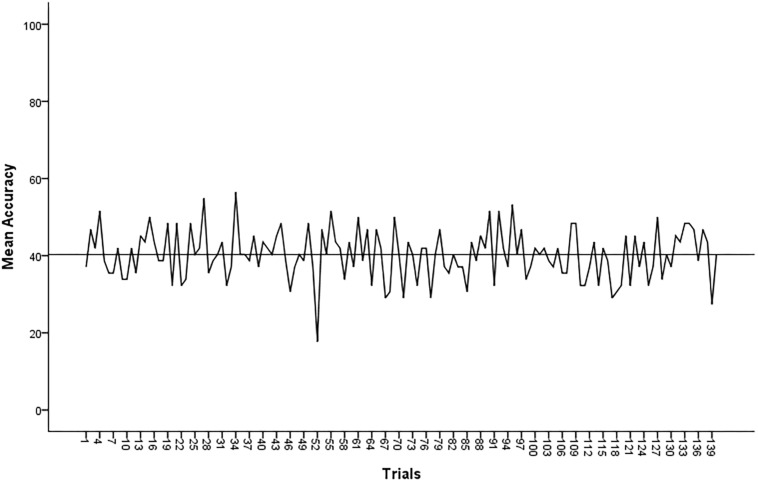
Accuracy average compiled for experimental task 2.

Regarding the average RT performance per group, no difference was found between men and women [*t*(29) = 0.028, *p* = 0.978], between distorted and undistorted groups [*t*(29) = 0.861, *p* = 0.396] and between satisfied and dissatisfied with body image groups [*t*(29) = −0.831, *p* = 0.413]. As presented in Figure 4, accuracy average throughout the trial sequence in this experiment is not crescent as it was observed in Experiment 1.

## Discussion

The experiments performed in this study required participants to discriminate general silhouettes (Experiment 1) and to judge whether the size of body silhouettes was similar in relation to their own body (Experiment 2). Accuracy rates in both tasks indicated that participants were able to recognize silhouettes at 17 ms, being more successful in the first task. Although previous literature has reported that at 17 ms few subjects were able to visually detect body stimuli (Pessoa et al., 2005), results from our study showed an overall accuracy of 63.9% for body silhouettes recognition considered alone. However, when asked to correctly judge the size of body silhouettes compared to their own body the accuracy rate dropped to 41.1%. In regard to visual clarity experience (PAS), scores from the first experiment suggested that visual experience was associated with the accuracy index, which is in agreement with previous results (Sandberg et al., 2010).

Results from the first experiment did not present effect considering explicit body image group criteria, which suggests that the attitude or perception toward own body does not produce performance differences for neutral stimuli discrimination. However, greater accuracy for the human silhouette recognition in the dissatisfied body image group is in agreement with evidences from attentional bias research that has shown higher levels of body stimuli recognition for dissatisfied body image groups in both clinical (Forghieri et al., 2016) and non-clinical settings (Glauert et al., 2010).

Yet this observed difference for body image groups was not confirmed in the context of body size judgment. Experiment 2 demonstrated that satisfied and dissatisfied body image participants had no differences in size judgments performance. Instead body image distortion served as a more differential explicit criterion. Subjects with higher rates of body image distortion presented greater accuracy for body silhouette recognition at 17 ms, with a tendency to better recognize thinner silhouettes. Such evidence suits as a better evidence of attention toward bodies, once it discriminates the direction of same class stimuli size recognition. This tendency was maintained when stimuli were inverted, which may signalize a top-down process. Differences in general performance between experimental blocks, considering the stimuli orientation, also reinforces the notion that a top-down process could be involved in the size judgment task. Stimuli set variations in size occur in a horizontal plane. By rotating the silhouette figures in 180° the size of the same stimuli set in regular position remained the same. So, if no human body feature identification had interfered in the size judgments, the rotation of stimuli would not had impacted the accuracy results. However, by inverting the stimuli set we have observed a reduction in participants’ accuracy rates.

To better discriminate thinner bodies had already been observed in non-clinical groups for intervals greater than 150 ms (Glauert et al., 2010; Joseph et al., 2016). However, results from these researches are contradictory since body dissatisfaction was negatively correlated with attentional bias toward thin bodies in one study (Glauert et al., 2010) and predicted attentional bias for thin bodies in the other study (Joseph et al., 2016). Differences may be explained by contrasting experimental paradigms applied, since exposure times to body silhouettes varied between 150 and 500 ms. In any case, our results did not replicate such effects once distortion of body image, not dissatisfaction, was the decisive discriminator factor for judgment performance. The lack of difference between satisfied and dissatisfied body image groups may suggest that this type of classification is more valid when comparing conscious body image assessment. This can be corroborated by the positive and significant correlations observed between attitudinal body image scores and the BSQ scores, which is in line with previous results (Fisher et al., 2019). Participants in the dissatisfied group presented higher BMI, which is also in agreement with previous research (Pull and Aguayo, 2011).

When accounting for the perceptual discrepancies between distorted and undistorted body image groups, it is interesting to note that Figure Rating Scales suggest that individuals who choose discordant figures from their actual body size as being their own body would have poorer decision making in perceptual body identification. Nevertheless, when comparing the accuracy rates specifically to their own body size figures the undistorted body image group had better scores than the distorted body image group, even though such difference was not significant. In this sense perceptual mistakes would be restricted to own body identification, but not to ideal silhouettes such as thinner bodies. However, more research should aim to better investigate such differences.

In regard to participants gender, in the first experiment no differences were found for accuracy rates. In the second experiment good accuracy rates were observed for men and women, although the latter presented greater general accuracy both in regular and inverted stimuli orientation. These results point to a gender difference in reference to body image assessment, which has been widely discussed in this literature (e.g., Muth and Cash, 2006; Alfano et al., 2011).

Responses for both tasks can be further discussed on what has been described as the “exposure effect” in visual perception literature. Usually, correct responses in recognition of neutral stimuli for brief interval exposure is increased over a number of trial sequence. Even in the absence of an explicit awareness of stimuli, discrimination still seems to get better over repeatedly exposure (Seamon et al., 1984). The most common interpretation for this phenomenon suggests that an affective mechanism operates a detection specification at the initial processing of visual information, aiding in the increase of accuracy through repetition. An alternative interpretation to the exposure effect is that for it to occur a previous awareness of the presented stimuli is necessary, which will account for a preference effect increasing accuracy (Zilva et al., 2013). Our results denoted an exposure effect solely for the first experiment, which contrasts to the previous stimuli awareness hypothesis. Participants in this study had already been exposed to the set of stimuli in Experiment 2 when responding to the FRS previously to experiments. Perhaps because the stimuli used in the second experiment were not exactly neutral, miscellaneous responses prevented a clear exposure effect. However, the absence of incremental accuracy in this experiment could be either explained by the lower accuracy rates observed compared to the first experiment rates. In any case more research should aim to investigate the exposure effect specifically for body silhouettes stimuli.

## Conclusion

Results indicated an inversion effect of silhouette figures recognition at 17 ms and differences of performance between distorted and undistorted body image groups. In addition, it has evidenced a specific effect of accuracy for silhouette figures thinner than the participants’ own body in the distorted body image group. These results may help to understand the contrast between attitudinal and perceptual aspects of body image at initial stages of visual processing. Implications could be extended to better refine the definition of body image perception and to further explore the associations between visual attention toward bodies and attitudinal/emotional components of body image. In line with continuous models of body representation and consciousness of the body, the present study offers evidence that fast body size judgment responses are partially associated with explicit body size judgments.

The present study had methodological limitations, such as the absence of a clinical comparison group, heterogeneous sample size groups between men and women, and the lack of a longer exposure interval to compare with the observed evidence at 17 ms. Further investigation should aim to compare temporal exposure intervals within implicit detection to look for perceptual differences regarding body size judgment in clinical groups. Also, it would be important to look for perceptual specificities in eating disorders population, once these groups could benefit from interventions focusing on implicit cognition. Considering that a specific trend toward thinner bodies was observed within distorted body image group it would be important to explore if such trend is also present in eating disorders population, specifically in anorexic patients.

Future studies should seek to continue refining experimental paradigms to investigate brief exposure intervals for body stimuli size judgment. Use of complex and simple body visual stimuli should also be addressed by comparing photograph stimuli to silhouette stimuli in rigorously controlled experiments.

## Data Availability Statement

The raw data supporting the conclusions of this article will be made available by the authors, without undue reservation, to any qualified researcher upon request.

## Ethics Statement

This study was carried out in accordance with the recommendations of the American Psychological Association. All subjects gave written informed consent in accordance with the Declaration of Helsinki and Brazilian regulation for studies with human participants (Resolution 510/2016 of the National Health Council). The protocol was reviewed and approved by the Ethics Committee from the Federal University of Rio Grande do Sul. All participants provided informed consent.

## Author Contributions

All authors conceived the research project, designed the experimental material, and contributed to the writing of the manuscript. AN and VE collected the data. TD conducted the analyses.

## Conflict of Interest

The authors declare that the research was conducted in the absence of any commercial or financial relationships that could be construed as a potential conflict of interest.

## Abbreviations

BMI: body mass index
BSQ: body shape questionnaire
FRS: figure rating scale
PAS: perceptual awareness scale.
